# Preliminary Antimicrobial Profile of *Solanum incanum* L.: A Common Medicinal Plant

**DOI:** 10.1155/2020/3647065

**Published:** 2020-01-21

**Authors:** Desta Berhe Sbhatu, Haftom Baraki Abraha

**Affiliations:** Mekelle Institute of Technology, Mekelle University, P. O. Box 1632, Mekelle, Ethiopia

## Abstract

Medicinal plants and plant remedies have been in use in Ethiopia for centuries. Studies on ethnobotany, ethnomedicine, and ethnoveterinary estimate that nearly 80% of Ethiopians use some type of medicinal plants and plant remedies. Medicinal plants are regarded as the most important and sometimes the only source of therapeutics in the country. Some 800 plant species are used as sources of medicine to treat about 300 physical and mental disorders. However, because these plant species are not adequately studied, there is a big limitation in their documentation, profiling, and management. Moreover, there is a continuous loss of knowledge about medicinal plants because the communities and people are adopting new lifestyles. Hence, this article reports the finding of a study aimed at providing the gross phytochemical characteristics and antimicrobial activities of ethanol and aqueous extracts of fruit, leaf, and stem of *Solanum incanum* L. against two Gram-negative (*Escherichia coli* and *Salmonella typhi*) and two Gram-positive (*Bacillus subtilis* and *Staphylococcus aureus*) bacteria for developing gross antimicrobial profile of the plant. Phytochemical screening of fruit, leaf, and stem extracts of *S. incanum* has shown that it is the source of alkaloids, saponins, flavonoids, glycosides, terpenoids, and steroids. According to agar disc-diffusion tests, 100 mg/mL extracts of the plant produced bacterial growth inhibition zones of 0.00 to 16.06 mm. Ethanol and aqueous leaf extracts produced inhibition zones ranging from 11.34 to 16.06 mm against all bacterial species. The greatest inhibition zone of 16.06 mm was recorded in *E. coli* subjected to ethanol leaf extract. The same extract resulted in a growth inhibition zone of 16.04 mm in *S. aureus*. The greatest growth inhibition zones in *B. subtilis* (13.34 mm) and *S. typhi* (11.56 mm) were observed with ethanol leaf and fruit extracts, respectively. Aqueous leaf extracts produced growth inhibition zones ranging from 10.45 mm (for *S. typhi*) to 14.02 mm (for *E. coli*). Ethanol leaf extracts resulted in the lowest Minimum Inhibition Concentration (MIC) of 1.56 mg/mL in *E. coli* and *S. aureus*. Therefore, fruits, leaves, and stems of *S. incanum* can be regarded as good sources of some bioactive compounds. The findings are important for taking measures for conservation and sustainable use of the plant as well as for further elucidation of its phytochemistry and antimicrobial efficacy of its constituents.

## 1. Introduction

Plants are the integral parts of human cultures in treating human and animal ailments. The worldwide share of plant-derived medicines for treating of human and livestock ailments is still massive [1]. For example, more than 800 plant species are used to treat nearly 300 physical and mental disorders in Ethiopia. The plants are the main and sometimes the only sources of medicine for approximately 80% of the people for centuries [2].

*Solanum* L. (*Solanaceae*) is an important plant taxon that comprises many multipurpose flowering plants in several communities and cultures. It has several species known for their medicinal importance [3]. *Solanum incanum* L. is one of the species with multiple traditional applications in many Ethiopian communities. The species is native to and widely distributed in the Horn of Africa. It has thorny leaves, yellow fruits, and blue flowers with yellow pistils [4]. It propagates by seeds, and the seeds germinate slowly. *S. incanum* is common around houses, overgrazed grasslands, wastelands, and road sides [5].

Fruits of *S. incanum* are found to be the source of several groups of phytochemicals, including terpenes, flavonoids, bioflavonoid, xanthenes, and multiple metabolites like tannins, saponins, cyanates, and oxalate, and anthraquinones [6] as well as steroid glycosides in the form of glycoalkaloids such as solanine and solasonine [7]. Likewise, the leaves of *S. incanum* L. are rich in minerals such as K [8] and Ca [9].

The plant is a good source of many phytochemicals used against pathogens and predators as well as for treating many human and animal ailments [7, 10, 11]. In Africa, it is used for treating angina, colic or indigestion, dandruff, fever, general infection, headache, liver pain, painful menstruation, skin diseases, snake bites, sore throat, stomach ache or abdominal pain, and wounds. Treatments or medications are done by drinking leaf, root and fruit decoctions, chewing and swallowing root sap, washing painful areas with leaf sap, and topically applying the plant's ash mixed with fat. Fruits and seeds of the plant are also used in curdling milk and making cheese [10, 12]. Besides, the plant is known for controlling cattle ticks and making compost [13]. Furthermore, fruit extracts of the plant have nematicidal compounds that can be used in managing of chilli root knot nematodes [14]. Antiprotozoal effects of *S. incanum* plant extracts were reported to be effective against *Plasmodium falciparum*, *Leishmania infantum*, *Trypanosoma brucei*, and *Trypanosoma cruzi* [15].

Majority of Ethiopian communities use traditional medicine. On the one hand, they have limited access and economic capacity to modern health care services. On the other, they have practical experiences and positive beliefs toward traditional medicine. For example, the authors of this article have known the fruits of *S. incanum* as source of extracts used for medicine, pesticides, soaps, and tanning. But the indigenous community and traditional healers believe that medicinal plants should be kept secret by the healers if they are to be effective. If the healers decided to reveal the knowledge, they often choose one curious and wiser family member and pass it on to her/him verbally. Such beliefs and practices have been the recipe for rapid loss of ethnobotanic, ethnomedicine, and ethnoveterinary knowledge before the scientific community makes adequate documentation and sharing. Moreover, despite the considerable contribution of traditional medicine to many Ethiopian communities and the urgent call by the scientific communities, there are not adequate efforts to transform the traditional indigenous practices and integrate the traditional medicine into modern health care system.

Efforts toward the collection of baseline data on medicinal plants for future phytochemical and pharmacological studies and innovation are very limited. Many scientists have underlined the urgent need for discovering new, safe, and cheap antibiotics with diverse chemical structures, novel chemical actions, and no adverse side effects in response to the emergence of resistant microbial strains to indiscriminate the use of antibiotics [16–19]. In this respect, medicinal plants with traditionally known bioactive constituents are important candidates for future pharmacological studies and drug discovery. Therefore, this article reports the findings of a study aimed at documenting the gross phytochemical profile and antimicrobial activities of fruit, leaf, and stem extracts of *S. incanum* as a contribution to future detailed scientific elucidation of its phytochemistry and biocidal properties.

## 2. Methods and Materials

### 2.1. Collection of Plant Materials and Extraction

Ripe fruits, mature leaves, and tender stems of *S. incanum* were collected from wild stands in gola'a-genahti area, northeast of Adigrat, Tigrai, and Ethiopia. Collection of plant materials by Ethiopian researchers for research and development purposes is granted by Article 15, Clause 1, of the Access to Genetic Resources and Community Knowledge, and Community Rights Proclamation of Ethiopia (No. 482/2006). The fruits were fully ripe yellow with no signs of browning. The leaves included stem tips with unopened leaves. The stems were prepared by removing the leaves and green, soft shoot tips. The fruits, leaves, and stems were cleaned off dust and dirt by washing with tap water. The fruits were sliced for drying. Then, all the plant materials were placed on laboratory bench top and air dried at room temperature. The dried plant materials were pulverized separately into powder with mechanical grinders and stored in airtight containers. The powdered contents were macerated with 80% v/v of ethanol and water for three days with occasional shaking and filtered to extract bioactive compounds. Upon observing the homogeneity of the contents, the ethanol and water-based maceration and filtration procedures were repeated three times to increase maceration and filtration efficiency. Then, the filtrates were lyophilized and kept for further work.

### 2.2. Phytochemical Analyses

Phytochemical analyses were performed on the lyophilized fruit, leaf, and stem extracts to identify the phytochemical constituents of the plant. The constituents were then tested using standard procedures for alkaloids, tannins, saponins, flavonoids, terpenoids, and steroids as summarized in Table 1 [20, 21].

### 2.3. Antimicrobial Activities of the Extracts

#### 2.3.1. Acquisition of Bacterial Strains

The antimicrobial activities of each plant extract were examined against two Gram-negative (*Escherichia coli* and *Salmonella typhi*) and two Gram-positive (*Bacillus subtilis* and *Staphylococcus aureus*). The strains were acquired from Tigrai Regional Health Laboratory and the Veterinary Microbiology Laboratory of the College of Veterinary Medicine at Mekelle University.

#### 2.3.2. Preparation of Nutrient Media

Bacterial colonies were inoculated into liquid nutrient broths and incubated in 200 rpm shaking incubator at 37°C overnight before the date of inoculation. Then, each broth culture was adjusted to match to McFarland 0.50 turbidity standard to get approximately 1 × 10^8^ CFU/mL. Likewise, Mueller–Hinton (M-H) media were prepared according to the procedures given by its manufacturer as growth media for agar disc-diffusion assay and Minimum Inhibition Concentration (MIC) test.

#### 2.3.3. Agar Disc-Diffusion Test

Susceptibility test was carried out using agar disc-diffusion method as outlined by Bari et al. with some modifications [21, 22]. Plates containing M-H agar media were prepared and kept overnight to ensure that the media are free of contamination. Side by side, 6.5 mm filter paper discs were prepared from sterile Whatman No. 1 filter paper using paper puncher. The M-H agar media plates were spread with 100 *µ*L of 12-hour-old bacteria cultures by spread plate technique and dried for minutes to let any surface moisture evaporate before applying the extracts. The filter paper discs were impregnated with 100 mg/mL concentration of *S. incanum* plant extracts. Then, the discs were placed onto the agar plates using forceps and allowed to diffuse for 1 hour at room temperature. For comparison, standard microbial agents—streptomycin (10 *μ*g) and gentamicin (10 *μ*g)—were tested along with the extracts. Three tests (replications) were conducted with each extract and test strain. Then, the plates were kept for two hours in the laboratory at room temperature and were transferred for incubation at 37°C for 24 hours. After incubation, the plates were observed, and the diameters of the zones of inhibition were measured using electronic digital caliper. Bacterial cultures with inhibition zone of greater than or equal to 7 mm diameter were considered as susceptible to extracts [23].

#### 2.3.4. Minimum Inhibitory Concentration (MIC) Tests

This test was carried out using serial broth dilution technique [21]. It indicates the lowest concentration of an antimicrobial agent that inhibits growth of a certain microorganism after 18 to 24 hours of incubation. The MIC tests were carried out for crude extracts that showed antibacterial activities based on the agar disc-diffusion test. Two-fold serial dilutions of 50 mg/mL of each crude extract were prepared to get concentrations ranging from 50 mg/mL to 0.195 mg/mL. The serially diluted extracts were added to 5 mL of liquid M-H media in test tubes. Then, 20 *μ*L of bacterial cultures were added to the tubes and incubated at 37°C for 24 hours. MIC results were studied through visual inspection and minimum concentration of the extracts that have shown no detectable growths were recorded as MIC.

## 3. Results and Discussion

### 3.1. Phytochemical Analysis

The phytochemical screening tests of the fruit, leaf, and stem crude extracts of *S. incanum* turned positive for alkaloids, saponins, flavonoids, glycosides, terpenoids, and steroids (Table 2). Many other groups of researchers had previously reported about the presence of glycosides, saponins, and steroids in the plant [4, 21, 24–27]. Aerial parts of the plant consist of two steroidal glycosidal alkaloids, solasonine and solamargine, and nonsteroidal components like three phenylalkanoic acids, benzyl-O-b-D-xylopyranosyl (1®2)-b-D-glucopyranoside, flavonoids, chlorogenic acid, adenosine, and new compound kaempferol 3-O-(6^2^¢-O-2,5-dihydroxycinnamoyl)-b-D-glucopyranosyl (1®2) b-D-glucopyranoside [15]. It is clear that the medicinal and bioactive properties and the effects of plants are affected by the quality and quantity of their chemical constituents. The medicinal and other bioactive characteristics of the extracts of *S. incanum* would be affected by the presence of all or some of these chemical constituents. The antibiotic actions of the fruits and leaves of the plant are attributed to their solanine contents and related glycoalkaloids [28–31]. Phytochemicals of several *Solanum* species, especially steroids, alkaloids, and saponins, are known for their cytotoxic and antimicrobial properties [4].

### 3.2. Antimicrobial Activities

Ethanol, methanol, and water are the most common polar extraction solvents for plant parts of *S. incanum* yielding antibacterial and antifungal phytochemicals [25–27, 32, 33]. Antimicrobial activities of ethanol and aqueous crude extracts of fruit, leaf, and stem of *S. incanum* were tested against standard strains of two Gram-positive and two Gram-negative bacteria. The crude extracts resulted in a varying degree of growth inhibition against the tested bacterial strains—ranging from no to outstanding growth inhibition. The aqueous stem extract showed no growth inhibition at all, whereas ethanol extracts of fruit, leaf, and stem yielded notable growth inhibition against the tested bacterial strain. Aqueous and ethanol leaf extracts were found to be more effective. For example, ethanol leaf extract resulted in growth inhibition of greater than 16.0 mm in diameter for *E. coli* and *S. aureus* (Table 3).

Ethanol and aqueous fruit extracts of the plant revealed some degree of antimicrobial activities only against the Gram-negative bacteria (i.e. *E. coli* and *S. typhi*). This could be attributed to the structural difference between Gram-positive and Gram-negative bacteria. The growth inhibition on the Gram-positive bacteria was below the standard inhibition zone for susceptibility. The difference in antimicrobial activities of the extracts may be accounted to differences in the amount and type of bioactive components, which in turn accounted to the source of extracts (i.e. plant parts). It is well established that the amount and types of bioactive compounds of plant extracts have different effects on different bacterial species. The presence of phytochemicals associated with insecticidal and deterrent activities in crude fruits extract of *S. incanum* was reported elsewhere [34]. The wider growth inhibition zone observed with the diluted ethanol and aqueous leaf crude extracts against all tested bacteria appear to imply that the leaves of the plant are the source of greater concentration of multiple phytochemical constituents. The fact that Ethiopian traditional healers often use leaf extracts in treating many skin and enteric infections e.g. [35–38] may have some relations to this observation.

Three of the tested bacteria exhibited some susceptibility to ethanol stem extracts of the plant except *S. typhi*. But the aqueous stem extracts did not indicate any inhibitive activities against all the bacteria. Higher microbial growth inhibitions were observed with ethanol extracts compared with aqueous extracts. This result may be attributed to the fact that 80% v/v ethanol can extract bioactive compounds with broad range of polarity, whereas water can extract compounds with strong polarity [39]. Moreover, the nature of the bioactive constituents may cause differential effects. In this regard, Cowan had confirmed that many plant bioactive compounds with antimicrobial activities are aromatic or saturated organic compounds soluble in ethanol [40]. Solvents have great effect on the extraction and efficacy phytochemicals [21]. In this case, ethanol is the most common solvent used in extracting plant bioactive substances for medical purposes. Our study has demonstrated that *S. incanum* stem may not be as good source of bioactive compounds compared with fruits and leaves. As one study with *Solanum marginatum* L. showed, stem extracts resulted in limited inhibitive activities against bacteria [41].

We observed that *E. coli* was the most susceptible species among the tested bacteria to *S. incanum* extracts. However, Alamri and Moustafa had reported findings contrary to ours where fruit extracts of the plant had profound antimicrobial activities against clinical isolates of *S. aureus* compared with *E. coli* and *Pseudomonas aeruginosa* [11]. The plant had resulted in strong antibacterial effects against *E. coli*, *Streptococcus pyogenes*, *S. aureus*, and *P. aeruginosa*. This is because each of the class of phytochemicals reported in this study contains several compounds that can affect a diversity of microorganisms in many ways [42].

The minimum inhibition concentrations (MIC) tests of the *S. incanum* were conducted with extracts that resulted in significant antimicrobial activities (with ≥7 mm inhibition zone) in the agar disc-diffusion tests. The data of our tests revealed that the MIC of the ethanol extracts ranged from 1.56 mg/mL to 50 mg/mL (Table 4). Ahmed had reported more or less similar observations [41]. Further purification of the crude extract may increase the growth inhibition capacity of the extracts.

## 4. Concluding Remarks

Many ethnobotanic, ethnomedicinal, and ethnoveterinary studies have reported that *S. incanum* is used in the treatment of many human and animal ailments e.g. [43, 44]. The gross phytochemical study of the extracts of fruit, leaf, and stem of the plant yielded results that strengthen the findings of previous studies e.g. [4, 21, 24, 28]. This study has also shown that aqueous and ethanol extracts of fruit, leaf, and stem of *S. incanum* have varying degrees of antimicrobial activities against four bacterial strains as reported by other researchers e.g. [11, 34, 41]. Although studies on the bioactivity of the plant often consider ripe fruits, our study established that leaf and stem extracts are also good sources of bioactive ingredients. Therefore, this study not only established the preliminary antimicrobial profile but also found out that leaves and stems are sources of antimicrobial agents. Our study lays the ground for further detailed studies to elucidate the chemistry of the bioactive constituents of the plant and their modes action as well as to ascertain their efficacy on multiple bacterial strains. The conservation and sustainable use of the plant is thus timely.

## Figures and Tables

**Table 1:** Phytochemical tests.

Tests	Procedures
Alkaloids	About half (0.5) gram of each crude extract was stirred in 5 mL of 1% aqueous HCl on a steam bath, cooled and filtered. Then, 1 mL of the filtrate was treated with a few drops of Wagner's reagent to observe the formation of yellow or brown precipitate
Flavonoids	Five to ten (5 to 10) drops of dil. HCl and small piece Mg powder was added to 0.5 mL of each extract and boiled for few minutes to observe reddish pink or brown color formation
Glycosides	Aqueous NaOH solution was added to each extract and was dissolved in 1 mL of water to observe the formation of yellow color
Saponins	About half (0.5) gram of each extract was mixed, shaken with distilled water in test tube to see the formation and persistency of froth for 15 minutes
Steroids	About 0.5 mL of each extract was dissolved in 5 mL of chloroform. Then, 5 mL conc. H_2_SO_4_ was added by sides of the test tube to observe turning of the upper layer to red color and H_2_SO_4_ layer to yellow with green fluorescence
Terpenoids	Five (5) mL of each extract was mixed in 2 mL of chloroform. Then, a layer was formed by adding 3 mL of conc. H_2_SO_4_ to observe reddish color formation in the interface

**Table 2:** Phytochemical screening of *S. incanum* crude extracts.

SN	Phytochemicals	Fruit extract	Leaf extract	Stem extract
1	Alkaloids	+	+	+
2	Saponins	+	+	+
3	Flavonoids	+	+	+
4	Glycosides	+	+	+
5	Terpenoids	+	+	+
6	Steroids	+	+	+

**Table 3:** Mean bacterial growth inhibition zones of agar disc-diffusion method treated with 50 mg/mL of *S. incanum* L plant extracts.

Plant parts	Agar disc-diffusion test	Mean of inhibition zone (in mm)			
		*S. aureus*	*B. subtilis*	*E. coli*	*S. typhi*
Fruit	Ethanol extract	6.90	6.95	13.15	11.56
	Streptomycin	18.75	16.78	18.11	20.25
	Gentamicin	15.73	15.68	17.82	21.32
	Aqueous extract	—	—	10.50	10.46
	Streptomycin	16.47	17.59	16.11	15.50
	Gentamicin	16.78	14.56	12.78	16.48

Leaf	Ethanol extract	16.04	13.34	16.06	11.34
	Streptomycin	16.02	15.60	16.02	10.61
	Gentamicin	15.03	14.98	15.03	11.00
	Aqueous extract	13.90	12.56	14.02	10.45
	Streptomycin	18.05	18.56	18.05	19.65
	Gentamicin	16.09	17.45	16.09	14.17

Stem	Ethanol extract	9.70	8.53	9.91	—
	Streptomycin	19.02	18.45	19.02	12.67
	Gentamicin	19.37	17.58	19.37	17.21
	Aqueous extract	—	—	—	—
	Streptomycin	13.83	14.35	13.83	15.38
	Gentamicin	15.66	14.91	15.66	19.24

**Table 4:** Minimum inhibition concentration of ethanol extracts of *S. incanum* L fruit, leaf, and stem extracts.

SN	Test strain	Extraction solvent	MIC of extracts in mg/mL		
			Fruit	Leaf	Stem
1	*B. subtilis*	Ethanol	25.00	6.25	25.00
		Aqueous	50.00	6.25	50.00
2	*E. coli*	Ethanol	3.13	1.56	12.50
		Aqueous	6.25	6.25	50.00
3	*S. aureus*	Ethanol	50.00	1.56	12.50
		Aqueous	50.00	6.25	50.00
4	*S. typhi*	Ethanol	12.50	12.50	50.00
		Aqueous	12.50	12.50	50.00

## Data Availability

The data used to support the findings of this study are available from the corresponding author upon request.
